# Digital Phenotyping: Ethical Issues, Opportunities, and Threats

**DOI:** 10.3389/fpsyt.2020.00473

**Published:** 2020-05-27

**Authors:** Giovanni Stanghellini, Federico Leoni

**Affiliations:** ^1^Department of Psychological, Territorial and Health Sciences, “G. d'Annunzio” University, Chieti, Italy; ^2^Center for Studies on Phenomenology and Psychiatry Medical Faculty, “D. Portales” University, Santiago, Chile; ^3^Department of Human Sciences, Verona University, Verona, Italy

**Keywords:** cause-effect relations, covariance, digital phenotyping, ethics, philosophy of psychiatry, prediction, technology

## Abstract

This paper explores the potential threats of digital phenotyping and the ways it may redesign our body experience and conceptualization. We argue that technology in digital medicine, and in psychiatry in particular, is not merely an *extrinsic* device to achieve improvements in knowledge, diagnosis, and treatment of diseases; rather, it *intrinsically* and unavoidably implies potential effects on what it is to be a human person, namely the embodiment and relatedness in human affairs, and not only in the clinical setting. Last but not least, digital phenotyping may improve prediction of abnormal behaviour, but not improve its causal explanation or psychological understanding.

## Introduction

Medicine is a knowledge and a technique of human bodies. Historically, it has been perhaps the most materialistic knowledge that mankind has developed and practiced, returning to us an image of man in its most concrete and terrestrial version. For better or worse, flesh and blood were the elements of medicine's concrete work, and the horizon of its overall vision of the human.

Medicine, and psychiatry as a part of it, have never been just a specialized science confined to diagnosing and treating diseases, but indeed a widespread set of apparatuses that shape our bodies, and decide what we can do with them or not, what we can expect from our lives or should not expect. In this sense, medicine has defined a whole field of possibilities and impossibilities of human existence, has imprinted large sectors of what is the contemporary form of Western life. Medicine has never been just a “local” science, but it has always proposed and sometimes imposed its explicit or implicit anthropology, whose ambitions and consequences have affected the entire scope of human life—even when it did not intend to do so, or when it expressly abstained from doing so.

## Dematerialized Medicine

It is not without the bewilderment of doctors as well as patients, that the object of medicine has recently *dematerialized*. Imaging techniques allow a new and increasingly refined approach to diagnosis, allowing areas of research and intervention unthinkable until a few years ago. They operate remotely thanks to a progressively extensive and powerful interface linked to the support of computing and the artificial intelligence resource. Digital phenotyping (1) is the emblematic example of an opportunity for extending our knowledge about the disorders that affect the human body, their course and outcome, and therefore it is a resource for diagnosis, especially early diagnosis; its version of tele-care is a means for monitoring patients, treating them timely and continuously over time (2).

There are several concerns about this approach, including ethical concerns which mainly focus on the most effective ways to preserve privacy (3). Another ethical issue is about the effects produced by technology on the patient-clinician relationship; this concern is usually counterbalanced by the argument that technology is seen as producing more improvements (e.g. precision diagnosis and treatments) than negative effects—the latter mainly confined to the worry that the interposition of technological devices may generate a quasi-dehumanized although effective practice (4).

## Digital Medicine Redesigns Our Bodies

A more subtle concern can be encapsulated in the following questions: is technology, like digital phenotyping, simply a “tool” to achieve improvements in medical practice? Is it an *extrinsic* device that has no effect on the way human beings experience and represent their bodies, interpersonal relationships, and the modes in caring about them and about human existence in general? Does technology *intrinsically* and unavoidably imply potential effects on what it is to be a human person, namely embodiment and relatedness in human affairs, and not only in the clinical setting?

Through technology, we have gained unprecedented access to our bodies and their functions, expanded our knowledge of their mechanisms, and the accuracy of our interventions on them. Yet—and here we come to our main concern—this means that through technology we are *redesigning our bodies*, and that through this set of tools and practices there will be new kinds of bodies, and new men and women too.

We must not think that these new techniques are a linear extension of the old techniques. Each new technique is a new trajectory of knowledge and intervention, only vaguely related to previous trajectories. No new technique is a linear extension of the previous ones, since no new technique applies to the same entities that were the object of their ancestors. Each new technique outlines a new field of unprecedented objects. Digital medicine does not operate in a new way on old bodies, rather it does new things on bodies that are also new. But the halo effect inhibits this implicit but powerful extension ranging from technical-specialistic innovation to the design of new forms of embodiment and of a new anthropology.

Let's take a simple and concrete example, that of the *drill*. Various paleoanthropological findings (5, 6) attest that this technology was available to our ancestors, and that sorcerers/doctors practiced interventions to the skull and perhaps to the brain. The drill-sorcerer/doctor has in front of him an object: solid, spatially discrete, stable over time. This will install an *epistemic polarity* of the type inside/outside, visible/invisible. This polarity implies a set of oppositions: hidden cause/visible effect. In medicine: etiology/semeiotics. For those who have a drill in their hand, diagnosis and treatment will mean first crossing a surface and accessing a profundity. Then, it will mean using what was previously invisible to causally explain the visible, since the inside is supposed to cause the outside. And, finally, it will mean to set forth to modify the inside/profound/invisible/cause/etiological in order to change the outside/surface/visible/effect/semiological. Each object is supposed to have other objects in its inside, and both knowing and intervening will mean handling from time to time the innermost object, the smallest element, the finest matter. Possibly, the ultimate objectivity, the tissue, the cell, the atom.

We are not arguing that the Neolithic surgeon was identical to the Renaissance surgeon or the contemporary surgeon. It may be that the Neolithic surgeon imagined that the object he was accessing was a spirit to be freed, more than a mood to drain, or a neoplasia to be removed. What matters is the structure of the *epistemic field* in front of which the three surgeons are located. The structure of the field does not vary at all with the changing meanings of the inside—be it a spirit, a mood, or a cell. As long as the technical instrument remains the drill, the structure of the field remains unchanged: inside versus outside, cause versus effect. This field-structure is entirely due to the nature of the technical instrument.

## Digital Medicine Looks for Covariance, Not for Causal Explanation and Psychological Understanding

The digital-clinician, as opposed to the drill-clinician, monitoring blood flow, oxygen consumption, the greater or lesser activation of certain vessels or brain areas, is in a quite different epistemic field. Where previously there was an *object*, now there is a *process*. The digital clinician is in the direct presence of a process. A process is not an object (spatially localized, discrete, and stable over time), but a set of fluctuations of a certain set of variables spatially diffused. In this new kind of epistemic field, diagnosis involves monitoring these variations of the process. To the digital-clinician, these variations are not exactly a hidden cause, an invisible etiology for the visible symptoms. The digital-clinician is not looking for causes hidden in the interior of a material body, rather he is studying the *covariance* of two sets of variables chosen for observation in a digitalized body. For example, a set of visual stimuli and a set of brain areas that activate to a greater or lesser extent. He will no longer be led to determine causes and effects.

Covariance aims to identify risk factors, not causes; and to allow prediction, not causal explanation and psychological understanding (see **Box 1**).

Box 1 **Risk factors are not causal explanation or understanding.**

*Case study 1* (smartphone-based empirical assessments of suicidal ideation): The aim of the study is to assess short-term variability in suicidal ideation in order to provide a novel method of improving the short-term prediction of suicidal ideation (7). Each day for 28 days, participants were signaled by a smartphone-based program at four random intervals separated by 4 to 8 hr (i.e., signal-contingent monitoring) to report on severity of suicidal ideation. The results of fine-grained examination of suicidal ideation advance the information of how suicidal ideation changes over short periods. Well-known risk factors for suicidal ideation such as hopelessness, burdensomeness and loneliness vary considerably over just a few hours and are correlated with suicidal ideation, but were limited in predicting short-term change in suicidal ideation.

*Case study 2* (fictional): Imagine that digital phenotyping through big data will allow us to predict that there is a covariance between increased suicidal behavior and increased consumption of, say, *soy milk* in the last 8 hr. Obviously there is no causal correlation between the two, yet psychiatrists may use this covariance as a predictor of suicidal intention without inquiring about causes and reasons of suicidality. It will be enough to determine a constant correlation between those two sets of variables in order to establish a prevention program. It may matter little to the digital-clinician why those sets of variables are varying together and according to which law. The fact that they vary together, and that you can write the formula of that covariance, is what matters.

The more data one collects (through digital phenotyping and big data), the less the causal paradigm will be important, and the more exhaustive the pure formulation of what might be called a *morphology* will be. Of course, not only explaining causally a given state will be less important, but even more so *understanding* the personal reasons for a given behavior, or how it feels, for a patient, to behave in a given way, will be less significant.

The digital-clinician may be led by his technological apparatus to abandon the idea that there are things in the world which act on other things, and may be tempted to embrace the perspective that in the world there are local fluctuations of a certain overall process. He will move along this epistemological slanted plane, not so much because he believes that reality is made of processes rather than of objects, but because the technique on which he relies upon reveals more about the processes and fluctuations than about the causal relations between objects (8, 9).

We all, doctors, non-doctors, and patients (10), are spellbound by the *screen* instead of the *drill*, and we will focus on dematerialized bodies, images, algorithms, processes, covariance, etc., rather than on physical bodies, words, personal stories, discrete events, causes and reasons, etc.

If this the trend of digital medicine is substituting cause-effect and motivational-psychological relations with relations of covariance, which effects will this trend have on therapeutic interventions? At present, therapeutic interventions are based on cause-effect relations in the sense that they try to target as much as possible on etio-pathogenic processes in order to eliminate their epiphenomena (namely, symptoms). It's hard to imagine what the interventions of digital medicine will be like. Devised to obtain a more accurate and comprehensive picture, a hypothesis about the outcome of digital medicine is that it will focus on epiphenomena, for instance abnormal behaviors, rather than on their biological or psychological etiology. This is perhaps too somber an outlook for the destiny of medicine—yet it seems to be a logical consequence of the epistemic field of digital phenotyping—focusing on covariance rather than etio-pathogenesis.

A final concern: will digital phenotyping help to distinguish normality from abnormality? This distinction—given the difficulty to differentiate the “normal” from the “abnormal” in a dichotomic way and given that the definition of “normality” is context-dependent and open to change—is at the moment based on constructs like dysfunction or suffering (11). If the trend is looking at a screen showing graphics and digits, the boundary between norm and pathology will be established numerically too. Will this produce arbitrary thresholds, as is the case for instance with borderline hypertension (12)?

Big data may produce a kind of *cyber-hypochondria*, that is the fear of being or getting sick based on an obsessive monitoring of one's own digitized bodily functions rather than on one's feelings of well-being or ill-being—another example of de-corporealization.

## Conclusions

In conclusion: in the face of such a radical transformation of techniques, it would be helpful to learn to do two things at the same time. On the one hand, we should learn how to take advantage of the instruments that contemporary technology provides for us, looking at the phenomena they show us and the possibilities of intervention that they open up. On the other hand, we should also learn to look at the instruments themselves, without being dazzled by the phenomena to which they seem to apply. When we worry that big data involves a privacy issue, it's already too late—even though we should worry about privacy. The real problem is not that we have to properly manage certain data about our bodies. The problem is that this data doesn't simply talk about how our bodies are made. They talk *about how our instruments* are made, and about *what our instruments can make of our bodies*.

An old proverb reads: when the wise man points to the moon, the fool looks at his finger. We could jokingly say that we are firmly convinced of the opposite: when the fool points to the moon, the wise man first looks at his finger.

## Author Contributions

GS and FL have contributed to this manuscript in equal parts.

## Conflict of Interest

The authors declare that the research was conducted in the absence of any commercial or financial relationships that could be construed as a potential conflict of interest.
